# A New Chanidae (Ostariophysii: Gonorynchiformes) from the Cretaceous of Brazil with Affinities to Laurasian Gonorynchiforms from Spain

**DOI:** 10.1371/journal.pone.0037247

**Published:** 2012-05-21

**Authors:** Cesar R. L. Amaral, Paulo M. Brito

**Affiliations:** Instituto de Biologia Roberto Alcântara Gomes, Universidade do Estado do Rio de Janeiro, São Francisco Xavier, Maracanã, Brazil; Raymond M. Alf Museum of Paleontology, United States of America

## Abstract

Based on specimens originally referred to as *“Dastilbe minor”*, a *nomem-nudum*, we describe a new genus of Chanidae †*Nanaichthys longipinnus* nov. gen. and sp. which exhibits several diagnostic characters such as the absence of orbitosphenoid and basisphenoid, anteriorly displaced quadrate-mandibular articulation, laterally expanded supraneurals, an acute angle between the preopercular limbs, expansion at the angle between the preopercular limbs, and a curved maxillary articular process. Its occurrence and supposed relationship within the Chanidae reinforce the influence of the Mediterranean Tethys over the Gondwanan main rift system prior to the Aptian/Albian highstands.

## Introduction

The breakup of the supercontinent Pangaea in the Middle Jurassic (∼175 Mya), creating the supercontinents Laurasia and Gondwana, and the subsequent breakup of Gondwana (∼90 Mya), were two of the most prominent paleogeographic events of the Phanerozoic.

Ever since the opening of the Caribbean Tethys and the establishment of at least intermittent connections between western Tethys and the eastern Pacific in the early Jurassic, a Tethyan fauna has had an influence in Gondwana. This influence is demonstrated by the occurrence and distribution of certain ammonites [1], bivalves [2], [3] and some genera of marine ichthyosaurs, plesiosaurs and crocodiles [4]–[6]. A similar influence is found during the Cretaceous when geodynamic processes related to the fragmentation of Gondwana continued to promote the dispersal of Tethyan fauna not only along the Central Atlantic, but also within the western Gondwanan rift system, as suggested by the distribution of foraminifera [7], [8], several actinopterygians (e.g. †Macrosemiidae, †Pycnodontiformes, †Ionoscopiformes, †Semionotiformes, †Aspidorhynchiformes, †Ellimichthyiformes, †Pycnodontiformes, †Ichthyodectiformes, and Gonorynchiformes [8]–[11].

Among these Tethyan forms, Gonorynchiformes [12] is a teleost fish clade represented by fossil taxa from Early/Late Cretaceous and Paleocene deposits from the Americas, Africa, Middle East, and Europe, and with extant forms distributed in marine waters of the Indo-Pacific and South Atlantic Oceans, and in several freshwater biotopes of Africa [13]–[15]. The clade comprises three extant families: Chanidae, Gonorynchidae, and Kneriidae, represented in the Cretaceous deposits of South America uniquely by Chanidae.

Chanidae are divided in two subfamilies, the extant Chaninae and the exclusively fossil †Rubiesichthyinae. Today Chaninae are represented by a unique extant species, *Chanos chanos* Forskal, 1775 [16], the “milk-fish” or “bango” and by the Gondwanan fossil genera †*Tharrhias* Jordan & Branner, 1908 [17], †*Dastilbe* Jordan, 1910 [18], and †Parachanos Arambourg & Schneegans, 1935 [19], and the Laurasian †Aethalionopsis robustus Traquair, 1911 [20], from the Early/Late Cretaceous of Brazil, Gabon, Equatorial Guinea, and Belgium respectively. The Laurasian †Rubiesichthyinae, until now represented by the genera †*Rubiesichthys* Wenz, 1984 [21] and †*Gordichthys* Poyato-Ariza, 1994 [22], have only been reported from the Early Cretaceous of Spain.

**Figure 1:**
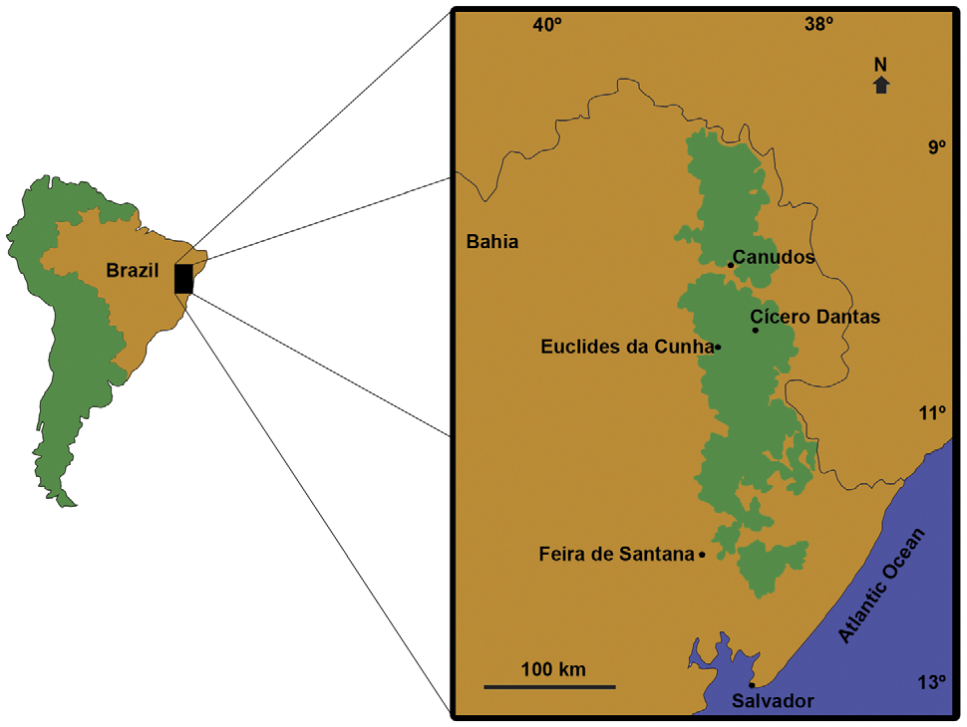
Map of the Tucano Basin, State of Bahia, Brazil, indicating the location of the fossils localities of the Marizal Formation in Euclides da Cunha and Cícero Dantas. Cretaceous strata in green.

Here we describe a new genus and species of Chanidae from the Cretaceous of Brazil and consider its affinities with the Laurasian †Rubiesichthyinae from the Early Cretaceous of Spain. The holotype was collected from the Marizal Formation of the Tucano Basin, Northeastern Brazil, one of the sub-basins of the Recôncavo-Tucano-Jatobá Rift-System. The Marizal Formation is dated as Early Cretaceous (Aptian?) mainly based on palynomorphs [23]. The specimens were collected in 1962 near Cícero Dantas, in the state of Bahia (Figure 1) and identified as “*Dastilbe minor”* [24], a *nomem-nudum* recently regarded as a synonym of †*Dastilbe crandalli* [25]. The fossils are permanently housed in the collection of the Divisão de Geologia e Mineralogia do Departamento Nacional de Produção Mineral, Rio de Janeiro, Brazil, and registered under the acronym DGM.

## Methods

All the specimens described here come from the same general locality near the town of Cícero Dantas, and are mainly preserved as negative impressions in hard siltstone. Latex peels were made from the fossils to obtain specimens with positive relief for detailed study. Silicone molds and then polyester resin casts of the original peels were made, because resin casts are more durable than latex peels, which tend to deteriorate within a short time. No specific permits were required for the described field work. In Brazil, the unique obligation we have is to contact the Departamento Nacional de Produção Mineral (DNPM–The Brazilian Geological Survey) explaining that we are doing fieldwork and after that to curate fossils in an official public collection, what is the case of the studied specimens deposited in the collection of the Departamento Nacional de Produção Mineral.

### Nomenclatural Acts

The electronic version of this document does not represent a published work according to the International Code of Zoological Nomenclature (ICZN), and hence the nomenclatural acts contained in the electronic version are not available under that Code from the electronic edition. Therefore, a separate edition of this document was produced by a method that assures numerous identical and durable copies, and those copies were simultaneously obtainable (from the publication date noted on the first page of this article) for the purpose of providing a public and permanent scientific record, in accordance with Article 8.1 of the Code. The separate print-only edition is available on request from PLoS by sending a request to PLoS ONE, 1160 Battery Street, Suite 100, San Francisco, CA 94111, USA along with a check for $ 10 (to cover printing and postage) payable to "Public Library of Science".

In addition, this published work and the nomenclatural acts it contains have been registered in ZooBank, the proposed online registration system for the ICZN. The ZooBank LSIDs (Life Science Identifiers) can be resolved and the associated information viewed through any standard web browser by appending the LSID to the prefix "http://zoobank.org/". The LSID for this publication is: urn:lsid:zoobank.org:pub:C750C1CE-1223-4378-843B-52A88B407B98.

## Results and Discussion

### Systematic Palaeontology

Ostariophysii *sensu* Rosen & Greenwood, 1970 [12].

Gonorynchiformes *sensu* Rosen & Greenwood, 1970 [12].

Chanidae *sensu* Poyato-Ariza, 1996 [26].

### *Nanaichthys* nov. gen

urn:lsid:zoobank.org:act:76B33275-4C05-4045-B482-F4D39BF6A7E8.

#### Derivation of name

*Nana*, named after ‘Nàná Burukù’, ancient Orisha, goddess of the muddy and primordial waters, plus *ichthys*, fish in Greek.

#### Diagnosis

As for the only known species below.

### *Nanaichthys longipinnus* nov. sp

urn:lsid:zoobank.org:act:E5A77C78-9E96-4AD9-8F8E-9CF5EFB9A96F.

#### Derivation of name

The specific epithet *longipinnus* refers to the eleven elongate anal fin rays exhibited by the holotype.

#### Holotype

**Figure 2:**
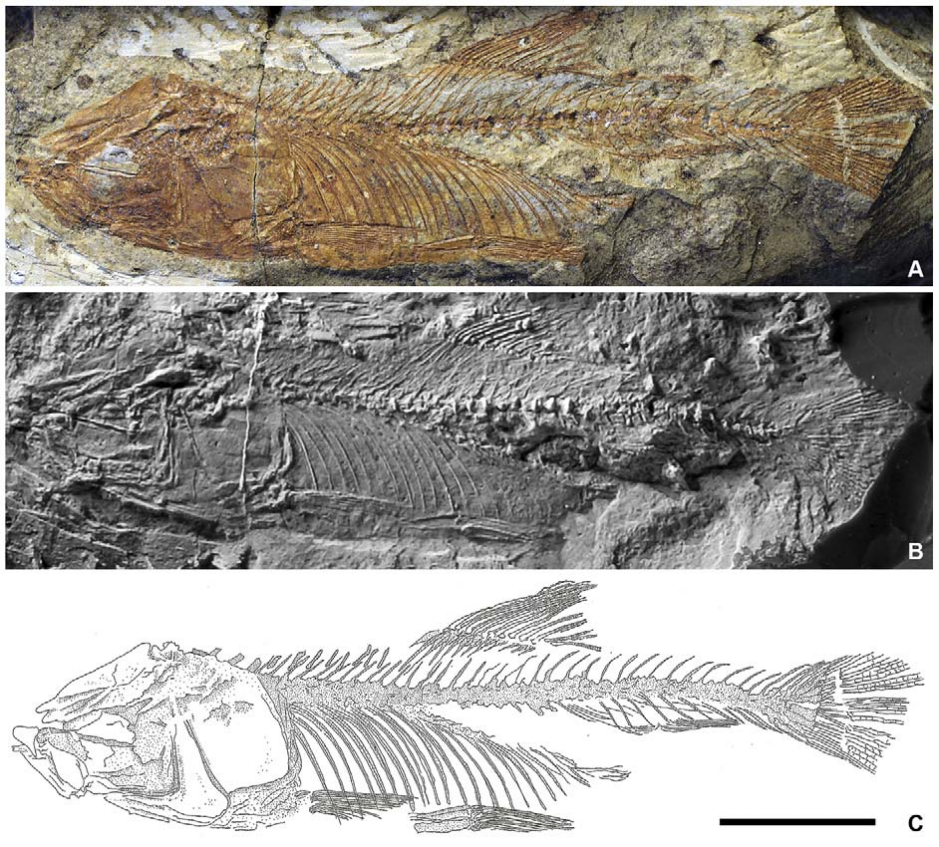
†*Nanaichthys longipinnus* nov. gen. and sp. Holotype DGM.1016-P. A) photograph; B) inversed peeling model; C) anatomical drawing. Scale bar equals 10 mm.

DGM.1016-P. Almost complete specimen, displaying its left side (Figure 2).

#### Referred specimens

**Figure 3:**
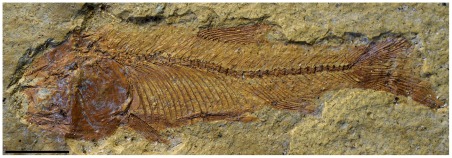
†*Nanaichthys longipinnus* nov. gen. and sp. DGM.535-P. Scale bar equals 5 mm.

DGM.1017-P, a collapsed and partially preserved specimen on its right side, and DGM.535-P (Figure 3), a partially preserved specimen on its left side which lacks the anterior part of the skull.

#### Horizon and type-locality

The specimens come from outcrops of the Marizal Formation of the Central Tucano Basin, near the entrance of Cícero Dantas city, State of Bahia, Brazil.

#### Diagnosis

Small-sized Chanidae, 48 mm total length (TL), with thirty-nine vertebrae; eleven anal fin rays, preopercule with an acute angle between horizontal and vertical processes; expanded posteroventral angle of the preopercular processes, and a small and curved articular process of the maxilla.

### Description

The holotype of †*Nanaichthys longipinnus* (DGM.1016-P) measures 48 mm total length (TL) and 43 mm standard length (SL). The skull length is about 35% of the SL. The mouth is edentulous, and the mouth cleft is anteriorly oriented. The maximum depth of the body is reached at the origin of the dorsal fin and is about 20% of SL. The dorsal fin originates in the middle of the SL, and the pelvic fin originates beneath the anterior half of the dorsal fin. The caudal fin is somewhat forked, with the caudal peduncle at about 10% of the SL.

**Figure 4:**
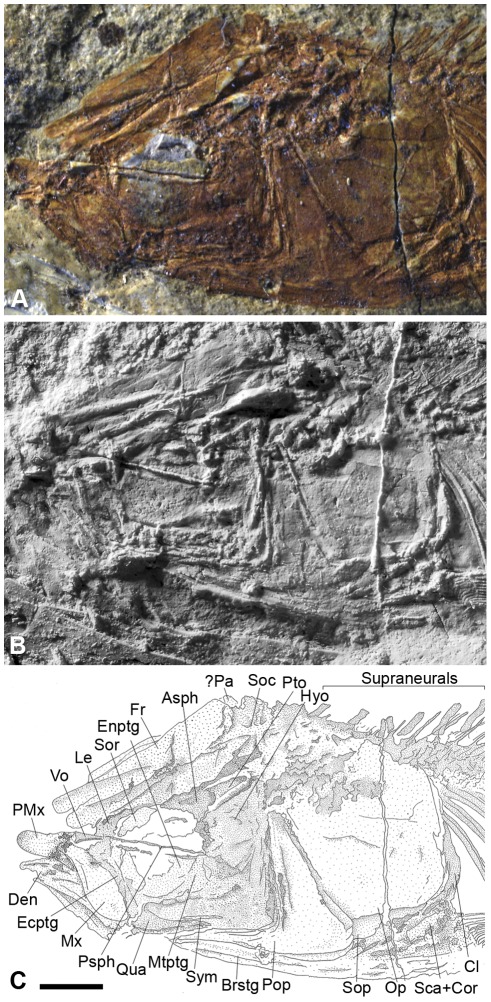
†*Nanaichthys longipinnus* nov. gen. and sp. DGM.1016-P, Holotype. A) photograph of the head region; B) inversed peeling model; C) anatomical interpretations. Abbreviations: Asph, autosphenotic; Brstg, branchiostegals; Cl, cleithrum; Fr, frontal; Hyo, hyomandibula; Den, dentary; Ecptg, ectopterygoid; Enptg, endopterygoid; Le, lateral ethmoid; Mtptg, metapterygoid; Mx, Maxilla; Op, opercle; Pa, left parietal; PMx, premaxilla; Pop, preopercle; Psph, parasphenoid; Pto, pterotic; Qua, quadrate; Sca+Cor, scapula + coracoids; Soc, supraocciptal; Sop, subopercle; Sor, supraorbital; Sym, sympletic; Vo, vomer. Scale bar equals 2 mm.

The frontals (Figure 4) are massive, forming the entire skull roof over the orbital region. They are wide through most of their length, with deep longitudinal grooves for the supraorbital sensory canal. The skull condition seems to be lateroparietal, and the frontals articulate posteriorly with the supraoccipital (Figure 4), which bears a small posterior crest. Laterally the frontals articulate with the marked pterotic and with the small and triangular autosphenotic (Figure 4). Anteriorly, the frontals articulate anterolaterally with the lateral ethmoids and anterolaterally with the supraorbital. A slender and edentulous parasphenoid (Figure 4) occurs in the middle of the orbital region. A small, triangular, and edentulous vomer (Figure 4) is present anterior to the parasphenoid.

The supraorbital (Figure 4) is partially preserved, exhibiting a broken lateral border. It is located anterolaterally to the frontals and seems to exhibit a concave-convex shape. Only the first infraorbital is preserved, as a thick and rectangular bone below the parasphenoid.

The jaws are edentulous. The premaxilla is concave-convex in shape (Figure 4) and exhibits the superior process broken, and a small oral process in contact with the maxilla. The maxilla (Figure 4) is club-like and presents a bulbous posterior region and a thin and small maxillary articular process. The dentary (Figure 4) is small and triangular, with a partially visible coronoid process reaching its maximum height in the middle of the dentary's length. The quadrate-mandibular articulation is anterior to the orbit. The quadrate (Figure 4) is triangular with an elongated posteroventral process. The sympletic (Figure 4) is small, slender, and posteriorly located with respect to the quadrate.

The hyomandibula (Figure 4) is robust, with a large dorsal head, an anteroventral process, and a small ventral process. The hyomandibula articulates with the neurocranium *via* a large facet comprising the autosphenotic and pterotic. Anteroventrally, the hyomandibula articulates with the wide metapterygoid (Figure 4). The endopterygoid (Figure 4) is partially preserved and seems to form the entire ventral wall of the orbit, articulating laterally with the slender ectopterygoid to delineate the orbital cavity.

The preopercle (Figure 4) is wide, with the preopercular sensory canal extending along its anterior border. Unlike the condition in other Chanidae (excluding members of the Rubiesichthyinae), this element exhibits an acute angle between horizontal and vertical processes and a long and narrow posteroventral expansion of the preopercular limbs. The horizontal process of the preopercle reaches the level of the middle of the orbit, not covering the quadrate-mandibular articulation.

The large and smooth opercle (Figure 4), corresponding to approximately one-third of the head length, articulates ventrally with the small and triangular subopercle (Figure 4) and anteriorly with the posterior border of the preopercle. The interopercle is present but almost totally covered and its shape cannot be determined in any of the specimens. Beneath the opercular series, the branchiostegals are visible; however, their number could not be determined precisely.

The pectoral girdle is robust and well ossified. The cleithrum (Figure 4) is triangular and posteriorly expanded, with the dorsal apex reaching the middle of the opercle dorsoventral height and the anterior apex reaching the postero-ventral expansion of the preopercle. The pectoral fin is formed by four radials and twelve segmented fin rays plus the first and small unsegmented ray. No post-cleithrum was found.

The well ossified pelvic bone is triangular, wide, and expanded where it articulates with the pelvic fin. The pelvic fin originates beneath the 18^th^ vertebra and is formed by five radials. We could not determine the number of pelvic fin rays in any of the specimens examined.

The dorsal fin originates above the 16^th^ vertebra and is formed by eleven segmented rays, with two smaller procurrent rays. The proximal pterygiophores are laterally expanded near the base of the dorsal fin. Small, halter-like distal pteriogiophores are present.

**Figure 5:**
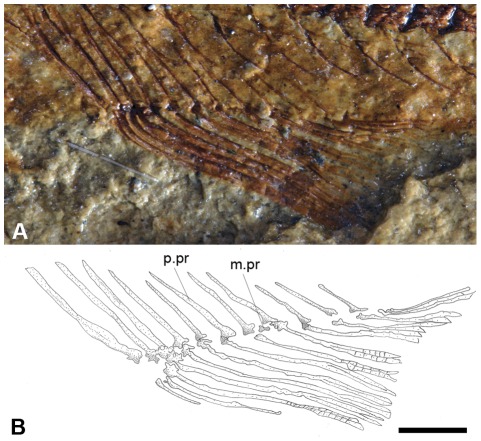
†*Nanaichthys longipinnus* nov. gen. and sp. DGM.535-P. A) photograph of anal fin; B) anatomical interpretation. Abbreviations: p.pr, proximal pterigiophore; m.pr, medial pterigiophore. Scale bar equals 1 mm.

The anal fin, as exhibited by specimen DGM-535-P, is located beneath the 26^th^ vertebra and is formed by elongate, laterally expanded proximal pterigiophores, small halter-like distal pterygiophores and eleven segmented and dichotomized fin rays (Figure 5), except for the small and unsegmented first ray.

Thirty nine vertebrae were observed in the specimens DGM1016-P and DGM-535-P, with the first four partially overlapped by the dorsal border of the opercle. Eighteen pleural ribs are preserved as slender bones delineating the abdominal cavity. The six anterior supraneurals (Figure 4) are laterally expanded, but we cannot precise if the first one exhibits a posterior expansion. Three sets of intermuscular bones were observed along the entire body. The epineurals are forked, and the epicentrals and epipleurals are small rod-like bones.

**Figure 6:**
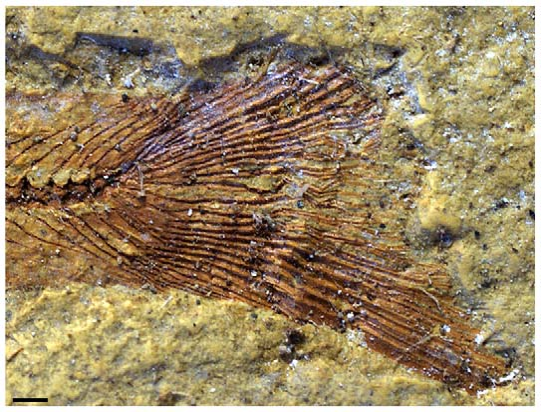
†*Nanaichthys longipinnus* nov. gen. and sp. DGM.535-P. Photograph of caudal fin. Scale bar equals 1 mm.

**Figure 7:**
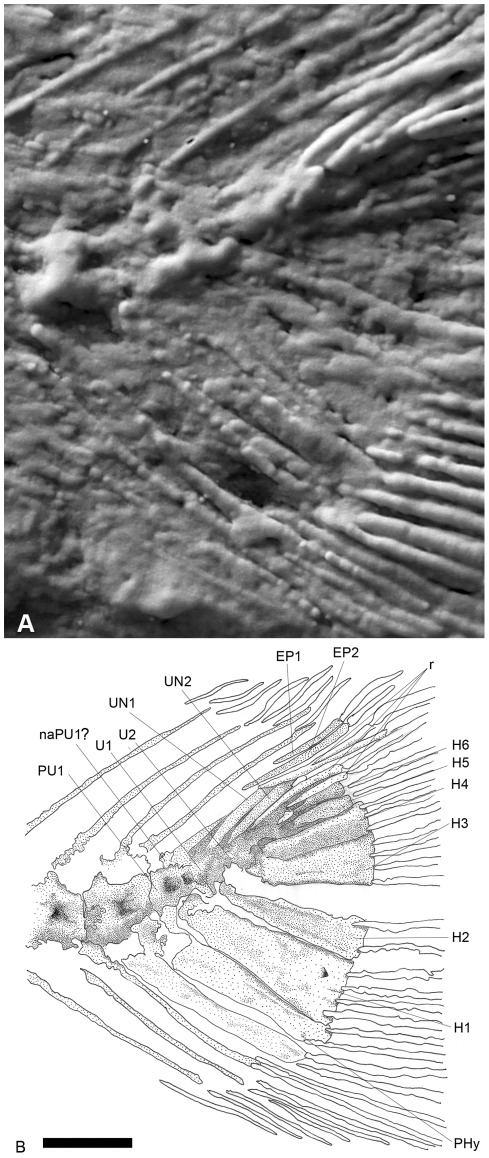
†*Nanaichthys longipinnus* nov. gen. and sp. DGM.535-P. A) inversed caudal fin peeling; B) anatomical interpretation. Abbreviations: PU1, preural centrum 1; UN1-2, uroneurals 1–2; EP1-2, epurals 1–2; H1-6, hypurals 1–6; PHy, parhypural; U1-2, ural centrum 1–2; naPU1?, neural arch of preural centrum 1; r, displaced caudal rays. Scale bar equals 1 mm.

The poorly preserved caudal fin of specimen DGM-535-P (Figures 6 and 7) seems forked. Its outline is formed by five dorsal and ventral unsegmented procurrent rays, reaching the level of the fourth preural centrum, and by twenty four segmented and dichotomizing rays (twelve for each lobe). The preural centra are fully ossified. The first preural vertebra is the first one to exhibit in its dorsal border a neural arch. Two independent ural centra, longer than high and smaller than the preural centra, seem to articulate with the second and third hypurals respectively. Six fan-shaped hypurals are present. Hypural 1, which is the largest of the series, is triangular and seems autogenous. The second hypural is smaller, half of hypural 1 width, and in contact with the first ural centrum. A diastema is present between the second and third hypurals. The hypural 3 is the large of the dorsal lobe and contact the second ural centrum. The anterior parts of the remaining three dorsal hypurals are poorly preserved, making interpretation of this region problematic. Two independent uroneurals are present but their posterior region is partially concealed by displaced fin rays. The first uroneural reaches the first preural centrum, and the second reaches the level of the first ural centrum. Two slender epurals were observed anterior to the first uroneural.

The scales are very small, cycloid, with small longitudinal striated marks. No lateral line scales were observed.

### Phylogenetics

We evaluated the phylogenetic relationships of this new taxon by a cladistic analysis based on a modified data matrix based on [15], with the inclusion of *Nanaichthys*. The modified matrix was run on PAUP 4b10 [27] with default options. We performed the same analysis performed by the previous authors but only with ACCTRAN optimization. Following Poyato-Ariza et al. [15] analyses, we excluded the taxa considered as problematic (*cf*. †*Apulichthys,* †*Erfoudichthys,* †*Halecopsis,* †*Leeceichthys,* and †*Sorbininardus*), and treated as ordered the characters 3, 8, 13, 15, 33, 57, 77, 78, 82, 95, 97, 99, 100, and 102. The following characters were modified from their original coding.

7. Brush-like cranial intermuscular bones: absent (0); present (1). [15] coded *Chanos chanos* as lacking brush-like cranial intermuscular bones. Following [28] and based on new specimens studied herein, *Chanos* was re-coded with the state (1).

13. Relative position of the parietals: medioparietals (in full contact with each other along their midline) (0); mesoparietal (1); lateroparietal (completely separated from each other by the supraoccipital) (2). Following [29] and the description for the Kneriidae provided by [15], the character was re-coded as non-applicable (−) for *Kneria* and *Parakneria*.

14. Parietal portion of the supraorbital canal: absent (0); present (1). Following [30] where the parietal branch of the supraorbital canal was observed to pierce the parietal in *Chanos*, the character was re-coded to state (1).

18. Mesethmoid: wide and short (0); long and slender, with anterior elongate lateral extensions (1); large, with broad posterolateral wing-like expansions (2); elongate and thin (3). Following [29], where variation in mesethmoid morphology of *Kneria* and *Parakneria* was described, we consider this character in need of further investigation and decided to delete it from our analysis.

47. Ossified interhyal: present (0); absent as an independent ossification (1). The charater was re-coded to state (1) in *Chanos*, *Kneria*, and *Parakneria* following [29], which reported its absence in all three genera.

53. Shape of opercular bone in lateral view: rounded/oval (0); triangular (1); squarish or square (2). We added the character state (2) for the square-shaped opercular bone for *Parakneria* and re-coded *Kneria* as polymorphic with the states (0) and (2) as reported by [29].

73. Second abdominal centrum: as long as first (0); shorter than first (1). [31] observed the variability of this character in the Kneriidae, presumably associated with sexual dimorphism. We consider this character in need of further investigation and therefore it was deleted from our analysis.

89. Lateral line and supracleithrum: supracleithrum pierced through dorsal region (0); supracleithrum pierced through all its length (1); lateral line does not pierce supracleithrum. The state was re-coded to state (2) for both *Kneria* and *Parakneria* following [29], which observed that the lateral line does not pierce the supracleithrum in either genus.

95. Neural arch and spine of preural centrum one: both well developed, spine about half as long as preceding ones (0); arch complete and closed, spine rudimentary (1); arch open, no spine (2). [29] observed variation of the character state in *Parakneria*. Therefore, following these authors, *Parakneria* was re-coded as polymorphic, exhibiting both states (1) and (2). *Chanos* was re-coded herein as presenting the state (1) based on observed specimens.

97. Number of uroneurals: three (0); two or one (1); none (2). This character was deleted from our analysis. No gonorynchiforms lack uroneurals.

106. Hypural 5 (plus 6 if present) and second ural centrum: separate (0); articulating (1). Homology problems emerge when considering the second ural centrum, which articulates with hypurals 3 and 4 as presented by [25] for the genus †*Dastilbe*, †*Tharrhias*, and the undescribed fossil Chanidae presented by [25], as homologous with the second ural centrum in †*Rubiesichthys* which articulates with the hypural 5. Among the studied taxa, the apomorphic state is found only in †*Rubiesichthys*. Regarding these problems we consider this character in need of further investigation and it was deleted from our analysis.

The character states for †*Nanaichthys* are presented in Table 1 and Table S1. For further discussion regarding the characters, see [15].

**Table 1:** Character states for †*Nanaichthys longipinnus*. All the characters presented here correspond to those presented by [15].

	1–5	10	15	20	25	30	35	40	45	50	55	60	65
*Nanaichthys*	11???	?0?00	002??	0??01	0?111	11100	?0001	10010	?0??0	1????	?1000	?2011	00000

	70	75	80	85	90	95	100	105	110	115	120	125	130
*Nanaichthys*	00?00	0????	??010	?1100	?0??0	01001	11000	00010	01000	?????	?????	?????	?????

**Figure 8:**
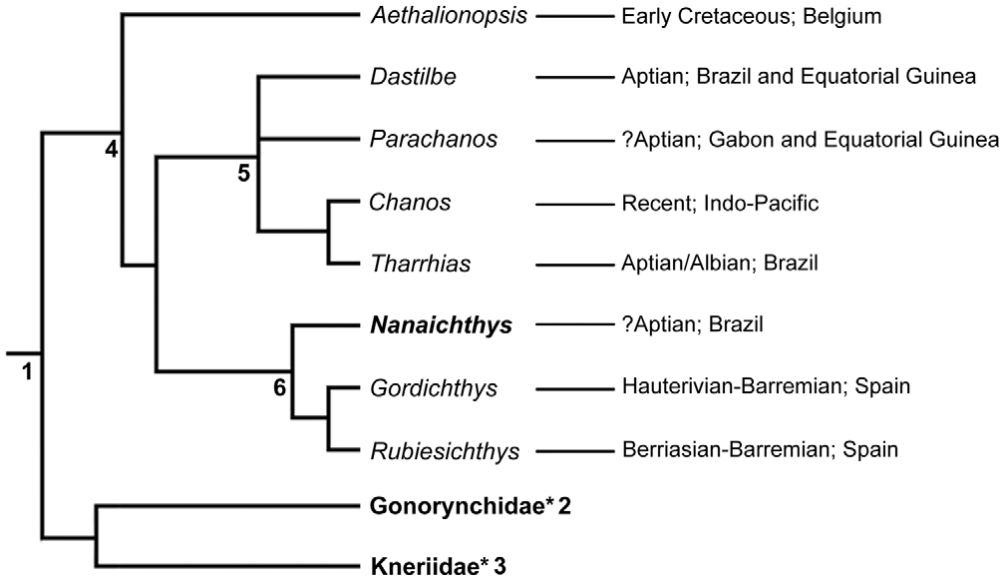
Area cladogram and strict consensus tree of the Chanidae based on 84 four most parsimonious trees with 233 steps (CI = 0.687, HI = 0.313 RI = 0.822, and RC = 0.564). Nodes 1) Gonorynchiformes; 4) Chanidae; 5) Chanini; 6) Rubiesichthyinae. Asterisks denote collapsed monophyletic clades not directly relevant to the discussion.

Our analysis running a heuristic search resulted in 84 most parsimonious trees with 233 steps (CI = 0.687, HI = 0.313 RI = 0.822, and RC = 0.564). The strict consensus of the obtained topologies (Figure 8) displays a total of 6 components. Here we consider the relationships of †*Nanaichthys w*ithin component 6, the †Rubiesichthyinae.

Poyato-Ariza [26] reviewed the Chanidae and diagnosed †Rubiesichthyinae as chanids which exhibit a strongly curved border of the maxillary articular process, a maximum height of the mandible reached at the central region, an acute angle between the preopercular limbs with a posterior expansion between the angle of the limbs, and a posterior process in the middle zone of the posterior border of the first supraneural. Recently Poyato-Ariza et al. [15] reviewed Gonorynchiformes interrelationships and re-diagnosed the †Rubiesichthyinae as chanids which additionally exhibit a small and flat nasal bone, a supracleithrum pierced through its entire length by the lateral line, and teeth on vomer and parasphenoid.

In our analysis, the †Rubiesichthyinae (Figure 8, Node 6) is diagnosed by 5 characters: (1) nasal bone small and flat, (2) curved dorsal and ventral border of the maxillary articular process; (3) acute angle formed by the preopercular limbs, (4) presence of a posterior process on the posterior border of first supraneural, and (5) lateral line and supracleithrum pierced all through this length. This result mostly agrees with ACCTRAN results from [15] except for character 42 (presence of teeth on vomer and parasphenoid), which was coded as absent in ours. In our analysis, the presence of teeth on the vomer and parasphenoid is autapomorphic for the group formed by †*Rubiesichthys* plus †*Gordichthys*, although the character's presence is still unknown for †*Rubiesichthys*.

Additionally, our result presents †*Nanaichthys longipinnus* as the most basal †Rubiesichthyinae, primarily on the presence of a curved dorsal and ventral border of the maxillary articular process and on the acute angle formed by the preopercular limbs. However, we note that the states for the remaining three characters of the †Rubiesichthyinae (cf. a nasal bone small and flat; the presence of a posterior process on the posterior border of first supraneural; and the lateral line and supracleithrum pierced all through this length) could not be observed in *Nanaichthys*. Therefore we regard this as an artifact of the ACCTRAN analysis and consider that these three characters should be better regarded as unique synapomorphies for the group formed by †*Rubiesichthys* and †*Gordichthys*.

Finally, †*Nanaichthys logipinnus* differs from *Dastilbe*, its previous generic designation, mainly by the acute angle formed by the preopercular limbs, the posterior expansion at the angle between the preopercular limbs, and the anal fin exhibiting 11 fin-rays.

### Biogeography

**Figure 9:**
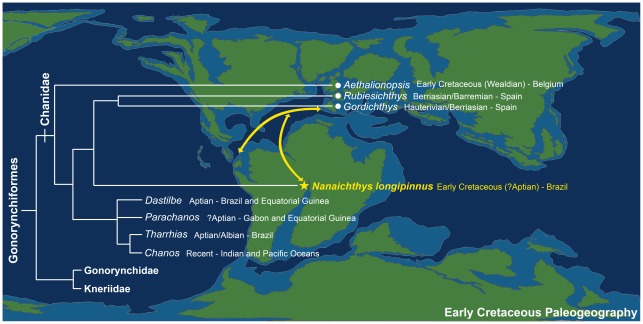
Area cladogram of the Gonorynchiformes and Early Cretaceous Paleogeography.

Although nowadays the spatial distribution of the extant Chanidae (cf. *Chanos chanos*) is exclusively related to the Indian and Pacific Oceans, the historical biogeography of the family seems to be related to the opening of the Caribbean/Mediterranean Tethys and the subsequent influx of the Tethyan fauna to several sedimentary basins related to the breakup of the supercontinent Gondwana (Figure 9).

The basal †*Aethalionopsis robustus* occurs in marine Early Cretaceous of Bernissart, Belgium. However, among the remaining subfamilies, except for the extant *Chanos chanos*, the Chaninae presents all other genera (cf. †*Tharrhias*, †*Dastilbe* and †*Parachanos*) in several Aptian/Albian rift-related basins from both Brazil and Africa (cf. Gabon and Equatorial Guinea). Previously the †Rubiesichthyniae was considered to have been restricted to Laurasian Early Cretaceous localities of Spain.

†*Nanaichthys longipinnus* is the first †Rubiesichthyinae described for western Gondwana. Among the †Rubiesichthyinae, †*Gordichthys* was described by [22] from the Hauterivian-Barremian of Las Hoyas, Cuenca, Spain, while †*Rubiesichthys* was described by [21] from the Berriasian-Valanginian of Montsec, Lérida, Spain, and has also been reported from the Hauterivian-Barremian of Las Hoyas by [32] and [33].

The occurrence of †*Nanaichthys* in the Aptian Marizal Formation of Brazil postdates the occurrence of the Mediterranean rubiesichthyans, therefore dating the divergence between them and †*Nanaichthys* at least as Berriasian-Valanginian. Therefore, the occurrence of †*Nanaichthys longipinnus* from the Marizal Formation suggests a faunal exchange not only from the Caribbean Tethys through the development of epeiric seas over the continental terrains of the Gondwana (e.g., [34]), but also between the Mediterranean Tethys and Laurasia, and the main Gondwanan rift system, at a time preceding the Aptian/Albian highstands.

### Conclusions

†*Nanaichthys longipinnus* nov. gen. and sp. exhibits several characters that support its inclusion within the Gonorynchiformes as well as its placement in Chanidae. These include the absence of an orbitosphenoid and basisphenoid, an antero-ventral expansion in the hyomandibula, a quadrate-mandibular articulation anterior to the orbit, an elongated symplectic, a concave-convex premaxilla, laterally expanded anterior supraneurals not in contact with each other, and the expanded opercle at about one-third of the head length.

Our results support the affinities between †*Nanaichthys longipinnus* and the Laurasian rubiesichthyns from Spain, mainly based on the small and curved articular process of the maxilla, the acute angle formed by the preopercular limbs, and posterior expansion at the angle between the preopercular limbs.

†*Nanaichthys longipinnus* differs from other †Rubiesichthyinae (cf. †*Rubiesichthys gregallis* and †*Gordichthys conquensis*) by presenting a combination of characters such as a gently (not strongly) curved maxillary process and the possession of eleven anal fin rays; from †*Gordichthys* in the body depth, vertebral count (39 for †*Nanaichthys* against 37 for †*Gordichthys*), and edentulous parasphenoid and vomer; and from †*Rubiesichthys* in body proportions, position of the dorsal, pelvic, and anal fins; and shape of the head, triangular in †*Nanaichthys* and elongated in †*Rubiesichthys*.

The proximity of †*Nanaichthys longipinnus* with the †Rubiesichthyinae from Spain reinforces the influence not only from the Caribbean Tethys but also from the Mediterranean Tethys and Laurasia prior to the Aptian/Albian marine transgressions over the continental terrains of Western Gondwana. This was probably influenced by the tectonically interconnected drainages that developed over the main rift axis prior to the well documented Aptian/Albian highstands.

## Supporting Information

Table S1 **Character states as presented by** [**15**] **with the inclusion of for †** ***Nanaichthys longipinnus*** **.**
(PDF)
