# Supplementary material for: A New Chanidae (Ostariophysii: Gonorynchiformes) from the Cretaceous of Brazil with Affinities to Laurasian Gonorynchiforms from Spain
Source: PLoS One. 2012 May 21;7(5):e37247. doi: 10.1371/journal.pone.0037247 (PMC3357423; doi:10.1371/journal.pone.0037247)
Supplement: Table S1 — Character states as presented by [15] with the inclusion of for † Nanaichthys longipinnus . (PDF) [file pone.0037247.s001.pdf]

Table S1. Character states as presented by [15] with the inclusion of for †*Nanaichthys longipinnus*.

	1	2	3	4	5	6	7	8	9	10	11	12	13	14	15	16	17	18	19	20	21	22	23	24	25	26	27	28	29	30	31	32	33	34	35	36	37	38	39	40	41	42	43	44	45	46	47	48	49	50	51	52	53	54	55	56	57	58	59	60	61	62	63	64	65
Diplomystus	0	0	0	0	?	0	0	0	0	0	0	0	0	0	0	0	0	0	0	0	0	0	0	0	0	0	0	0	0	0	0	0	0	0	0	0	0	0	0	0	0	0	0	0	0	0	?	?	?	?	?	0	0	0	0	0	0	0	0	0	0	0	0	0	0
Brycon	0	1	0	0	0	0	0	1	0	1	0	0	2	0	0	0	0	0	0	0	0	0	0	0	0	0	0	1	0	0	0	0	0	0	0	0	0	0	1	0	0	0	0	0	0	0	0	0	0	0	0	0	0	0	0	0	0	0	0	0	0	0	0	0	0
Opsariichthys	0	1	0	0	0	0	0	1	0	0	0	0	0	0	0	0	0	0	0	1	0	0	0	0	0	0	0	1	0	0	0	0	0	0	0	0	0	0	1	0	0	0	0	0	0	0	0	0	0	0	0	0	0	0	0	0	0	0	0	0	0	0	0	0	0
Aethalionopsis	?	?	?	?	?	?	?	?	0	0	0	?	2	1	1	0	?	0	?	1	0	?	1	?	0	1	1	1	0	0	?	0	0	0	1	1	0	0	?	0	0	?	?	?	0	?	?	?	?	?	?	1	0	0	0	?	1	0	1	1	1	0	0	0	0
Chanos	1	1	1	1	1	2	1	1	0	0	0	0	2	1	2	1	0	2	0	1	0	0	1	1	0	1	1	1	0	0	1	0	0	0	1	1	1	0	1	0	0	0	0	2	0	1	1	1	0	0	0	1	0	0	0	0	1	0	0	2	1	0	1	0	0
Charitopsis	1	1	?	?	?	?	?	?	1	0	0	?	2	?	1	?	?	0	0	1	0	?	0	1	0	1	0	1	1	0	0	?	?	0	0	?	?	0	?	1	2	0	0	1	?	0	?	?	?	?	?	0	1	1	0	?	0	0	0	0	0	1	0	0	0
Charitosomus	1	1	2	0	?	1	1	?	1	0	0	0	2	0	1	0	0	0	0	1	0	?	0	1	0	1	0	1	1	0	0	?	2	0	0	0	0	0	1	1	2	0	0	1	1	0	?	?	?	?	?	0	1	0	0	?	0	0	0	0	0	1	0	0	0
Cromeria	1	1	2	0	0	1	0	2	0	1	0	0	2	0	3	0	1	1	1	1	0	0	0	1	0	0	0	1	1	0	0	1	0	1	0	2	2	0	1	0	3	0	?	?	1	2	1	1	1	1	1	0	0	0	0	1	0	0	0	0	0	0	0	0	2
Dastilbe	1	1	1	0	0	?	?	?	0	0	0	0	2	0	1	1	0	0	?	1	0	?	1	1	0	1	1	1	0	0	1	0	0	0	1	1	0	0	1	0	0	?	?	?	0	1	?	?	?	?	?	1	0	0	0	?	1	0	1	0	1	0	1	0	0
Gonorynchus	1	1	2	0	0	1	1	1	1	0	1	1	2	0	1	0	0	0	0	1	1	1	0	1	0	0	0	1	1	1	0	1	0	0	0	0	0	1	1	1	2	0	0	1	1	0	0	1	1	0	1	0	1	0	0	1	0	0	0	0	2	0	0	0	0
Gordichthys	1	1	?	0	?	?	?	0	0	0	0	0	1	0	1	0	?	0	?	1	0	?	1	1	1	0	1	1	0	0	1	0	0	0	1	1	0	0	1	0	0	1	?	?	0	1	?	?	?	?	?	1	0	0	0	?	2	0	0	1	1	0	0	0	0
Grasseichthys	1	1	2	0	0	0	0	2	0	1	0	0	2	0	3	0	1	1	1	1	0	0	0	1	0	0	0	1	1	0	0	1	0	0	0	1	0	-	1	0	3	0	0	2	1	2	1	1	1	1	1	0	0	0	0	1	0	0	0	0	0	0	0	0	2
Hakeliosomus	1	?	?	0	0	?	?	?	1	0	0	0	2	0	1	0	?	0	0	1	?	?	0	1	0	1	0	1	0	0	0	?	1	0	0	0	0	0	1	1	2	0	0	2	1	0	0	?	?	?	?	0	1	0	0	?	0	0	0	0	0	0	0	0	0
Judeichthys	1	1	?	0	?	1	1	?	1	0	0	0	2	0	1	0	?	0	0	1	0	?	0	1	0	1	0	1	1	0	0	?	1	0	0	?	0	0	1	1	2	0	0	2	1	?	?	?	?	?	?	0	1	0	0	?	0	0	0	0	0	0	0	0	0
Kneria	1	1	2	0	0	1	0	0	0	0	0	0	-	-	3	0	1	1	1	1	0	0	0	1	0	1	0	1	1	0	0	1	0	0	0	0	0	0	1	0	2	0	1	2	1	2	1	1	1	1	1	0	?	0	1	1	0	0	0	0	2	0	0	0	0
Notogoneus	1	?	2	0	0	1	1	?	1	0	0	0	2	0	1	0	0	0	0	1	?	?	0	1	0	?	0	1	1	1	0	?	0	0	0	?	0	?	?	1	2	0	0	1	1	0	?	?	?	?	?	0	1	0	0	?	0	0	0	0	0	0	0	1	0
Parachanos	?	?	?	0	?	?	?	1	0	0	0	0	2	0	1	?	?	0	?	1	0	?	1	1	0	1	1	1	0	0	?	0	0	0	1	1	0	0	?	0	0	?	?	?	0	1	?	?	?	?	?	1	0	0	0	?	1	0	1	1	1	0	1	0	0
Parakneria	1	1	2	0	0	1	0	0	0	0	0	0	-	-	3	0	1	1	1	1	0	0	0	1	0	1	0	1	1	0	0	1	0	0	0	0	0	0	1	0	2	0	1	2	1	2	1	1	1	1	1	0	2	0	0	1	1	0	0	0	2	0	0	0	0
Phractolaemus	1	1	2	0	0	1	0	1	2	0	0	0	2	0	2	0	1	0	1	1	0	0	0	1	0	0	0	1	0	0	0	1	0	0	2	2	2	0	1	0	2	0	2	2	0	2	0	1	1	0	1	0	0	0	0	1	0	1	0	3	1	0	0	0	1
Ramallichthys	1	1	?	0	0	1	1	?	1	0	0	0	2	0	1	0	?	0	0	1	0	?	0	1	0	1	0	1	1	0	0	?	1	0	0	?	0	0	1	1	2	0	0	2	1	?	?	?	?	?	?	0	1	0	0	?	0	0	0	0	0	0	0	0	0
Rubiesichthys	1	1	?	0	?	?	?	0	0	0	0	0	2	0	1	0	?	0	?	1	0	?	1	1	1	0	1	1	0	0	1	0	0	0	1	1	0	0	1	0	0	?	?	?	0	1	?	?	?	?	?	1	0	0	0	?	2	0	0	1	1	0	0	0	0
Tharrhias	1	1	1	0	1	?	0	1	0	0	0	0	2	0	1	1	0	2	?	1	0	?	1	1	0	1	1	1	0	0	1	0	0	0	1	1	1	0	1	0	0	0	0	2	0	1	?	1	?	?	?	1	0	0	0	?	1	0	1	2	1	0	1	0	0
Nanaichthys	1	1	?	?	?	?	0	?	0	0	0	0	2	?	?	0	?	?	0	1	0	?	1	1	1	1	1	1	0	0	?	0	0	0	1	1	0	0	1	0	?	0	?	?	0	1	?	?	?	?	?	1	0	0	0	?	2	0	1	1	0	0	0	0	0

	66	67	68	69	66	66	66	66	74	75	76	77	78	79	80	81	82	83	84	85	86	87	88	89	90	91	92	93	94	95	96	97	98	99	100	101	102	103	104	105	106	107	108	109	110	111	112	113	114	115	116	117	118	119	120	121	122	123	124	125	126	127	128	129	130
Diplomystus	0	0	0	0	0	?	0	0	0	0	0	0	0	0	0	0	0	0	0	0	0	0	0	0	0	0	0	1	0	0	0	0	0	0	?	0	0	0	0	0	0	0	0	0	0	0	?	?	?	?	?	?	?	?	?	?	?	?	?	?	?	?	?	?	?
Brycon	0	0	0	0	0	0	1	0	1	0	0	1	0	0	0	1	2	0	0	1	0	0	0	0	0	0	1	0	1	1	0	1	3	1	2	0	0	1	0	0	0	0	0	0	0	0	0	0	0	0	1	1	0	0	0	0	0	0	1	0	1	0	0	0	1
Opsariichthys	0	0	0	0	0	0	1	0	1	0	0	1	0	0	0	1	2	0	0	1	0	0	0	0	0	0	1	0	1	1	0	1	3	1	0	0	0	0	0	0	0	1	0	0	0	0	0	0	1	0	0	0	1	0	0	0	0	0	0	0	0	0	0	1	0
Aethalionopsis	0	0	0	0	0	0	?	?	?	?	?	?	?	1	1	0	?	?	0	?	?	0	?	?	0	0	0	1	0	1	0	0	?	1	0	0	0	0	1	0	0	1	0	0	0	?	?	?	?	?	?	?	?	?	?	?	?	?	?	?	?	?	?	?	?
Chanos	0	0	1	0	0	0	1	0	1	1	0	1	0	1	0	0	1	1	0	0	0	0	1	0	0	0	1	1	1	1	0	1	3	1	1	0	0	1	1	0	0	1	1	0	0	0	0	0	1	0	1	1	1	0	0	0	0	0	0	0	0	0	0	0	0
Charitopsis	0	0	1	1	?	1	1	0	?	?	?	2	2	?	0	?	1	1	0	?	?	?	1	?	0	0	1	0	?	?	?	?	?	1	?	?	?	?	?	?	?	?	0	0	?	0	?	?	?	?	?	?	?	?	?	?	?	?	?	?	?	?	?	?	?
Charitosomus	0	0	1	1	0	0	1	0	1	1	?	2	2	?	0	?	1	1	0	1	0	0	1	0	0	0	1	0	1	1	0	1	3	1	2	1	1	2	0	0	0	1	0	0	?	0	?	?	?	?	?	?	?	?	?	?	?	?	?	?	?	?	?	?	?
Cromeria	0	1	?	1	0	0	1	0	1	1	1	1	1	0	0	1	1	1	0	-	0	1	1	0	0	0	1	0	1	2	0	2	3	2	0	1	0	1	1	0	0	0	0	1	-	0	0	0	1	0	1	1	1	0	0	0	1	0	0	1	0	1	0	0	0
Dastilbe	0	0	?	0	0	0	1	0	1	?	0	1	0	1	0	0	1	1	0	0	0	0	0	0	0	0	1	0	0	1	0	1	1	0	0	0	0	0	1	0	0	1	0	0	0	0	?	?	?	?	?	?	?	?	?	?	?	?	?	?	?	?	?	?	?
Gonorynchus	0	0	2	1	2	1	1	0	1	1	0	1	1	0	0	1	1	1	0	1	0	0	1	0	1	0	1	0	1	1	0	2	3	2	2	1	1	2	0	0	0	0	0	0	1	1	0	1	1	0	1	1	1	0	0	0	0	1	0	0	0	1	1	0	0
Gordichthys	0	0	?	0	0	0	1	0	?	?	?	1	0	1	0	?	1	?	0	0	1	0	0	1	0	1	1	0	0	1	0	1	1	0	0	0	0	0	1	0	1	1	0	0	0	0	?	?	?	?	?	?	?	?	?	?	?	?	?	?	?	?	?	?	?
Grasseichthys	0	1	?	1	0	0	1	0	1	1	1	1	1	0	0	1	1	0	0	0	0	1	1	0	0	0	1	0	1	1	0	2	3	2	0	1	0	2	1	0	0	0	0	1	-	0	0	0	1	0	1	1	1	0	1	-	?	-	-	-	0	1	0	0	0
Hakeliosomus	0	0	1	1	0	0	1	0	1	?	?	2	2	?	0	?	1	1	0	1	0	0	1	?	0	0	1	0	1	1	0	1	3	1	2	1	0	2	0	0	0	0	0	0	?	0	?	?	?	?	?	?	?	?	?	?	?	?	?	?	?	?	?	?	?
Judeichthys	0	0	1	1	0	0	1	0	1	?	?	2	2	?	0	?	1	1	0	1	0	0	1	0	0	0	1	0	1	1	0	1	3	?	2	1	?	2	0	0	0	1	0	0	?	0	?	?	?	?	?	?	?	?	?	?	?	?	?	?	?	?	?	?	?
Kneria	0	1	1	1	0	0	1	0	1	1	1	1	1	0	0	1	1	1	1	0	0	1	1	2	0	0	1	0	1	1	0	2	3	2	0	0	0	1	1	0	0	0	0	0	0	0	0	0	1	1	1	1	1	0	0	0	1	0	0	0	0	1	0	0	1
Notogoneus	0	0	1	1	?	1	1	?	1	1	?	0	0	0	0	1	1	1	0	0	0	0	1	0	0	0	1	0	1	1	0	1	3	0	0	1	0	0	1	0	0	1	0	0	1	0	?	?	?	?	?	?	?	?	?	?	?	?	?	?	?	?	?	?	?
Parachanos	0	0	?	0	0	0	?	?	1	?	?	?	?	1	0	?	1	?	0	?	?	0	0	0	0	0	1	?	0	1	0	1	2	0	0	0	0	0	1	0	0	1	0	0	?	?	?	?	?	?	?	?	?	?	?	?	?	?	?	?	?	?	?	?	?
Parakneria	0	1	1	1	1	0	1	0	1	1	1	1	1	0	0	1	1	1	0	0	0	1	1	2	0	0	1	0	1	?	0	2	3	2	0	0	0	1	1	0	0	0	0	0	0	0	0	0	1	1	1	1	1	0	0	0	1	0	0	0	0	1	0	0	0
Phractolaemus	0	1	1	2	0	0	1	1	1	1	0	1	1	0	0	1	1	1	0	1	0	1	1	0	0	2	1	0	1	2	0	2	3	2	0	1	0	1	0	1	0	0	0	0	0	0	1	0	1	0	1	1	1	1	0	1	1	0	0	0	0	1	0	0	?
Ramallichthys	0	0	1	1	0	0	1	0	1	?	?	2	2	?	0	?	1	1	0	1	0	0	1	0	0	0	1	0	1	1	0	1	3	1	2	1	1	2	0	0	0	1	0	0	?	0	?	?	?	?	?	?	?	?	?	?	?	?	?	?	?	?	?	?	?
Rubiesichthys	0	0	?	0	0	0	1	0	1	?	?	1	0	1	0	?	1	?	0	0	1	0	0	?	0	0	1	0	0	1	0	1	1	0	0	0	0	0	1	0	0	1	0	0	0	0	?	?	?	?	?	?	?	?	?	?	?	?	?	?	?	?	?	?	?
Tharrhias	0	0	1	0	0	0	1	0	1	1	0	1	0	1	0	0	1	1	0	0	0	0	0	0	0	0	1	0	0	0	1	0	1	0	0	0	0	0	1	0	0	1	0	0	0	0	?	?	?	?	?	?	?	?	?	?	?	?	?	?	?	?	?	?	?
Nanaichthys	0	0	?	0	0	0	?	?	?	?	?	?	0	1	0	?	1	1	0	0	?	0	?	?	0	0	1	0	0	1	1	1	0	0	0	0	0	0	1	0	0	1	0	0	0	?	?	?	?	?	?	?	?	?	?	?	?	?	?	?	?	?	?	?	?
